# Field demonstration of irradiated sodium alginate as tea production booster

**DOI:** 10.1016/j.heliyon.2020.e05881

**Published:** 2021-01-04

**Authors:** Mohammad Afzal Hossain, Jahid M.M. Islam, Md. Mozammel Hoque, Shamsun Nahar, Mubarak A. Khan

**Affiliations:** a Department of Food Engineering and Tea Technology, Shahjalal University of Science and Technology, Sylhet 3114, Bangladesh; b Institute of Radiation and Polymer Technology, Bangladesh Atomic Energy Commission, P. O. Box-3787, Dhaka, Bangladesh; c Environmental Biotechnology Division, National Institute of Biotechnology, Savar, Dhaka, Bangladesh; d School of Science, Monash University Malaysia, Bandar Sunway, Subang Jaya, Malaysia

**Keywords:** Sodium alginate, Tea production, Growth promoting activity, Gamma radiation, Natural growth promoter

## Abstract

Sodium alginate oligomers were tested for tea plant growth promoter and anti-fungal agent in this experiment. Sodium alginate solutions were irradiated by Co-60 gamma radiation with different radiation doses to produce the oligomers. Irradiated solutions were then diluted into 150, 300 and 500 ppm prior to foliar application. Solutions were applied through foliar spraying at 7 days interval and the best response of tea plants in terms of various attributes were recorded. Tea buds were collected in 10 days of interval and the growth attributes like- total number of buds, fresh weight of buds, average leaf area and weight per bud, weight of made tea etc. were calculated. The experiment was continued up to 12 weeks and the attributes were averaged to get results per plucking. 12 kGy radiation doses along with 300ppm solution showed the best results and about 36% increase in productivity was found based on the fresh weight of buds. Total fungal count in tea leaves was also found to be reduced greatly. Based on the present study, irradiated sodium alginate could be used as safe and environmentally friendly agent to increase tea production.

## 1 Introduction

Numerous health benefits and refreshing characteristics are leading increased tea (*Camellia sinensis* L.) consumption all over the world but its production has not been increased to that extent (Ahmad and Hossain, 2013; Ahmad et al., 2013a, 2016). Application of chemical fertilizers and fungicides may increase the production of tea, but their applications are hazardous for overall environment including animals and plants. It is approved that continuations of these perilous practices lead to destruction of soil structures, agro-ecosystem disorder and overall loss of biological diversity (Jagtap et al., 2018). In this regard, adopting eco-friendly cultivation practices is necessary to achieve sustainable yield increase.

Some active chemical compounds present in the entire or in specific parts of the plants or insects have been widely used for therapeutic activity or beneficial effects (Dey et al., 2019; Ahmad et al., 2013b) from which plant growth promoting activity is a notable one. These plant or insect produces are eco-friendly and cost effective. A lot of studies have reported growth promoting activity of chitosan and alginate on various plants (Parvin et al., 2013; Liu et al., 2019; Ahmed et al., 2020). However, very few studies have been conducted on tea plants. Hossain et al. (2013) studied the effect of gamma radiation treated chitosan on tea plant growth but there is no study on the effect of alginate.

Sodium alginate is naturally occurring biomaterials extracted from sea weeds like brown algae (Ikeda et al., 2000). It is a gum, extracted from the cell walls of brown algae. The chemical compound sodium alginate is the sodium salt of alginic acid. Its empirical formula is NaC_6_H_7_O_6_. In extracted form it absorbs water quickly; it is capable of absorbing 200–300 times of its own weight in water (Roew, 2009). To enhance its biological activity alginate is often degraded to produce alginate oligosaccharide having low molecular weight and higher functional groups (Shabbir et al., 2017; Salachna et al., 2018; Ali et al., 2014). A wide range of physical, chemical and biological methods have been routinely applied to produce this alginate oligosaccharide, which often lead to high degree of physico-chemical heterogeneity in output (Liu et al., 2019; Hu et al., 2013; Chandía et al., 2004). Among these methods ionizing radiation processing like Co-60 gamma radiation is one of the most convenient and efficient methods (Ali et al., 2014; Ibrahim et al., 2014). Gamma radiation can modify the viscosity, molecular weight, hydrophilic and mechanical properties of sodium alginate resulting in enhanced properties. Previous work focused on foliar application of irradiated and radiolytically degraded sodium alginate showed significantly increases the growth, photosynthesis, physiological activities, and alkaloid production *in Catharanthus roseus* L. (Idrees et al., 2011; Naeem et al., 2015). Uddin et al. (2020) reported a significant increase of pineapple size, sweetness, aroma, and flavor in case of the mixed treatment by sodium alginate and chitosan at 30 days of interval. Irradiated sodium alginate solution (ISAS) was also found effective in case of other plants like: *Mentha arvensis* L. (Naeem et al., 2012), *Acokanthera oblongifolia* Hochst (Taha and Sorour, 2019), *Artemisia annua* L. (Aftab et al., 2016), etc.

It is thought that the plants may have the capacity to recognize the specific natural polysaccharides oligomers, which further stimulates the growth, development, shoot elongation and defense responses of plants. It is however, considered that specific structural and size requirements exist to induce the physiological processes of these oligosaccharides in plants (El-Mohdy, 2017). Interestingly, the mechanism by which the degraded polysaccharides (oligomers) stimulate the growth and development processes in different plants still in vogue and needs further investigations. However, Idrees et al. (2011) suggested that irradiated sodium alginate treated plants trapped more sunlight and thus promoted photosynthesis and growth as well. Aftab et al. (2016) also found that the application of irradiated sodium alginate improved the total chlorophyll content in *Artemisia annua* L. and thus improved the plants growth.

In the study of Hossain et al. (2013), it was found that radiation processed chitosan increased the productivity of tea plants about 38% and reduced the total fungal counts dramatically. To our knowledge, no similar study was conducted on tea plants for growth promotion by ISAS. Taking into consideration, this research work was undertaken to evaluate the effect of radiation processed sodium alginate on tea plants at different radiation doses and concentrations and to make a comparative study among treated and untreated plants.

## 2 Materials and methods

### 2.1 Preparation of sodium alginate solution

Sodium alginate was supplied by Merck, Germany and used without further purification. 2% sodium alginate solution was prepared using distilled water as solvent. Solution was made in room temperature through continuous stirring by magnetic stirrer.

### 2.2 Radiation processing

Sodium alginate solution was irradiated by Co-60 gamma radiation at different doses (8, 12, 16, 20 and 24 kGy) with a dose rate of 4.5 kGy per hour. Radiation induced chain scission of the alginate polymer chain and produced low molecular weight alginate with lesser monomer units. The gamma radiation plant was situated in Institute of Radiation and Polymer Technology, Bangladesh Atomic Energy Commission, Savar, Dhaka, Bangladesh.

### 2.3 Measuring the viscosity of irradiated sodium alginate solution

Viscosity of alginate solutions was measured by Ubbelohde glass capillary viscometer (Model 1 Type B J-Sil) at 25 °C. At first, density of sodium alginate solution was measured by using a specific gravity bottle. Then the reservoir bulb of the Ubbelohde viscometer was filled up with sodium alginate solution and drawn to the measurement bulb by rubber tube suction. The rubber tube was sealed in order to prevent the solution from falling back to the reservoir. The liquid was then allowed to free fall by releasing the rubber tube. The flow time of the solution from the top to the bottom marking of the measuring bulb was noted. The same was done for pure distilled water to aid in calculation. The viscosity of sodium alginate was measured by using the following formula (Equation 1):

(1) $$\text{Viscosity of sodium alginate }\left(\eta_{s}\right)=\frac{\eta_{w}\times \rho_{s}\times t_{s}}{\rho_{w}\times t_{w}}$$

Where,

$\rho_{s}$ = Density of sodium alginate
$t_{s}$ = Flow time of sodium alginate solution
$\eta_{w}$ = Viscosity of water at 25 °C
$\rho_{w}$ = Density of water
$t_{w}$ = Flow time of water

### 2.4 Experimental design for field trial

A plot area of 03-years-old BT-2 variety tea plantation was used for this experiment. It was carried out at Ali Baher Tea Estate, Sylhet, Bangladesh (24°55′N latitude, 91°51′E longitude, and 30 m altitude). The soil analysis of the experimental plot showed that it has a pH of 4.7, which is in the range of optimum pH for tea soil. Organic matter and Nitrogen content were 1.63 and 0.09%, respectively. Amount of Potassium, Magnesium and Calcium was 0.04, 1.16, and 4.72 mEq/100 g of soil, respectively. The phosphorous, Sulphur, Iron, Aluminum, and Zinc concentration were 1.3, 28.5, 13.32, 43.2, and 0.43 ppm, respectively. All of these parameters are in suitable range for tea production. Each treatment was replicated three times, and each replicate had five plants. Two different factors namely- radiation doses and concentrations of the alginate solutions were taken into consideration for the filed trial of irradiated sodium alginate solutions (ISASs). No other chemical was used in the experimental plot during the study period. Plants were grown under naturally incited environmental conditions.

### 2.5 Application of irradiated sodium alginate solution (ISAS) on tea plants

The ISASs were diluted at different concentration level before spraying. Each solution was diluted to 150, 300, and 500 ppm using double distilled water. Hence, radiation doses used for alginate solutions were 0 (un-irradiated), 8, 12, 16, 20, and 24 kGy. Thus, the total numbers of solution prepared from 2% sodium alginate solution were 18 (6 × 3 = 18). The solutions of different doses were applied at the rate of 200 mL per experimental plot using normal hand sprayer at every 7 days interval.

### 2.6 Evaluation of growth promotion of tea plants

Tea buds were plucked with 10 days of interval for 12 weeks. The growth attributes like- total number of buds, fresh weight of buds, average leaf area, weight per bud and weight of made tea were determined by averaging the data found from each plucking round and represented by attributes per plucking. Leaf area for secondly leaves of arbitrarily selected buds was measured using portable laser leaf area meter (CI-202, CID Bio-Science, USA). Made tea was prepared by traditional orthodox black tea manufacturing procedure (Pou et al., 2019).

### 2.7 Evaluation of fungal resistance of the treated tea plants

The fresh tea buds from different treatment plots were collected and soaked into 0.89% saline water at 1g/9mL ratio. The mixture was shaken well and 100 μL of leaf free mixture was inoculated onto potato dextrose agar (PDA) media and spread using L-shaped spreader. PDA petri dishes were then incubated at 30 °C for 3 days. The enumeration of fungal count was done by colony counter.

### 2.8 Data analysis

The data was analyzed statistically using MiniTab-19 statistical software (Minitab, LLC, State College, Pennsylvania, USA). Descriptive statistics such as mean and standard error of the results was displayed. One-way Analysis of Variance (ANOVA) was used to observe the mean differences of different responses against different concentrations and radiation doses of ISASs. Finally, Tukey multiple comparison test was applied to determine the significant mean differences. All tests were done using 5% significant level (*p* < 0.05).

## 3 Results

### 3.1 Viscosity of the irradiated solution

Viscosity of ISAS is directly related to the molecular weight alginate. This means, viscosity of alginate solution should be lower if there is any radiation induced chain scission of alginate polymeric structure. The results obtained from the viscosity measurement are shown in Table 1. It was found that the viscosity of sodium alginate solution showed decreasing trend with increased radiation doses. This suggested that alginate polymeric chains were broken with radiation dose and thus produced low molecular weight alginate (Parvin et al., 2013). This size reduction of alginate might lead to increased absorption and functionality after foliar application of the solution.

**Table 1:** Changes in viscosity of Na-alginate solution with different radiation doses.

Radiation Doses	Control	08 kGy	12kGy	16 kGy	20 kGy	24 kGy
**Viscosity (centipoise)**	1.1948	1.1846	1.0982	1.0336	1.0278	1.0048

### 3.2 Effect of ISAS on growth promotion of tea plants

**Figure 1:**
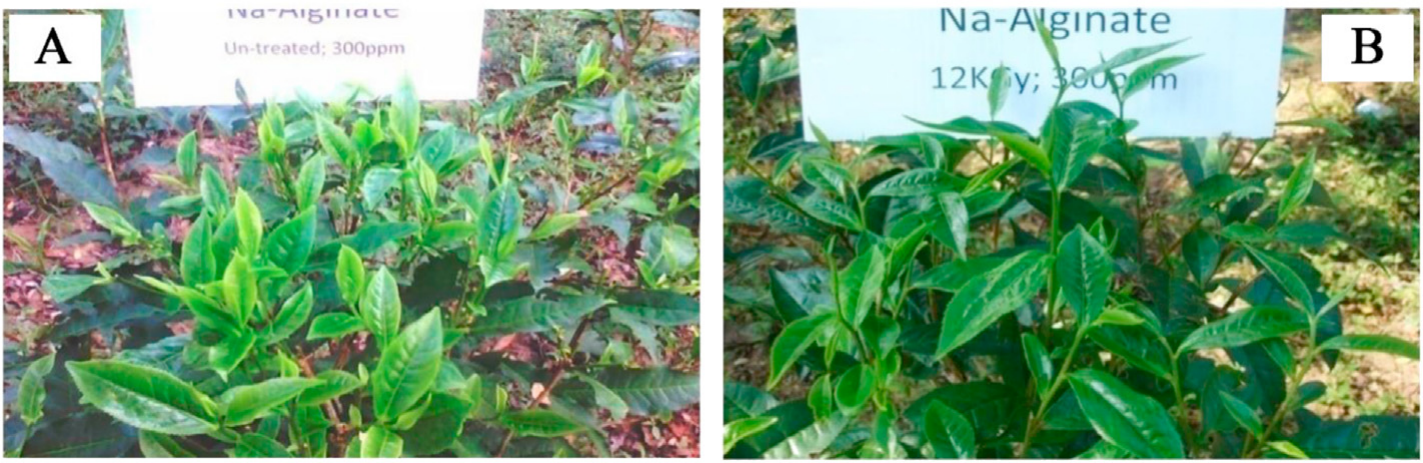
Effects of irradiated sodium alginate solution on tea plants; A. 300 ppm un-treated alginate solution applied and B. 300 ppm 12 kGy gamma radiation treated alginate solution applied.

ISAS of different concentrations were sprayed to the experimental plots once a week and studied for growth promotion parameter variations. Overall tea production was increased in all of the radiation treated sample sprayed plants compared to the untreated control (Figure 1). Different indicators of growth promotion are presented below.

#### 3.2.1 Number of buds

**Figure 2:**
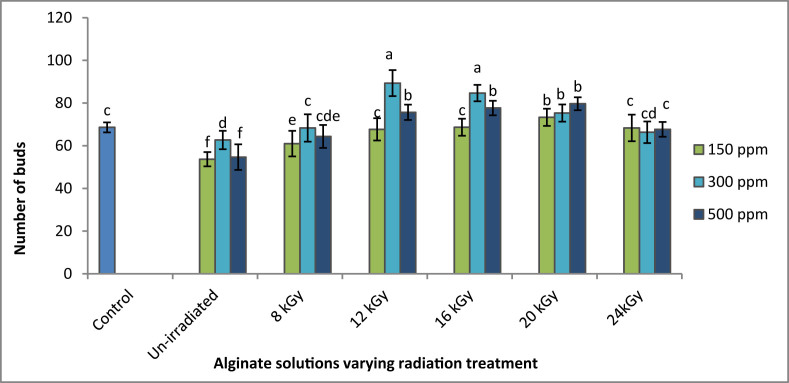
Effects of irradiated sodium alginate solution on number of buds of tea plants. ∗Means ± Standard Error of Means those do not share a letter are significantly different (P < 0.05) according to Tukey Method.

The ISAS treatments showed significant impact on number of buds of experimental plants which is represented in Figure 2. It exhibits that maximum bud numbers obtained when tea plants were treated by 300 ppm ISAS (12 kGy radiation treatment). After 12 kGy radiation dose, the bud numbers were gradually decreased at higher radiation doses. At both low and high concentrations of ISAS, bud numbers also decreased.

#### 3.2.2 Weight of Fresh Buds and made tea

Table 2 represent data of the average weight of collected tea buds and made tea from the experimental plots. The highest amount of fresh tea leaves was obtained from the tea plants treated with 12 kGy gamma irradiated sodium alginate solution at 300 ppm concentration. Statistical analysis showed that the average weight of tea bud collected from 12kGy, 300ppm alginate solution treatment (36.538 ± 0.173 g of fresh tea leaves per plucking round) was significantly different from other treatments. It was found that maximum 35.71 % increase in the fresh weight of tea buds was obtained compared to the untreated control.

**Table 2:** Effects of various doses of gamma radiation treated sodium alginate solutions on weight (g) of fresh buds and made tea.

Radiation Doses	Weight of Fresh Buds (g)			Weight of Made Tea (g)		
	Concentration			Concentration		
	150 ppm	300 ppm	500 ppm	150 ppm	300 ppm	500 ppm
0 kGy	24.084 ± 0.197^K^∗	31.006 ± 0.203^DE^	22.958 ± 0.190^L^	5.920 ± 0.104^**ij**^∗	6.892 ± 0.185^**cd**^	5.290 ± 0.205^**k**^
8 kGy	20.642 ± 0.337^M^	31.608 ± 0.428^CD^	25.352 ± 0.245^J^	4.830 ± 0.302^**l**^	7.216 ± 0.125^**c**^	6.054 ± 0.212^**hij**^
12 kGy	27.332 ± 0.422^GH^	36.538 ± 0.173^A^	27.634 ± 0.149^GH^	5.906 ± 0.220^**ij**^	8.626 ± 0.140^**a**^	6.558 ± 0.235^**defg**^
16 kGy	28.974 ± 0.563^F^	32.146 ± 0.296^C^	28.574 ± 0.287^F^	6.340 ± 0.195^**fghi**^	6.818 ± 0.234^**cde**^	6.776 ± 0.132^def^
20 kGy	30.664 ± 0.426^E^	28.578 ± 0.265^F^	34.578 ± 0.128^B^	6.918 ± 0.096^**cd**^	6.556 ± 0.245^**defg**^	7.890 ± 0.245^**b**^
24 kGy	26.136 ± 0.207^I^	27.772 ± 0.374^G^	25.002 ± 0.538^J^	6.208 ± 0.142^**ghi**^	6.430 ± 0.189^**efgh**^	5.688 ± 0.145^**jk**^
Control	26.922 ± 0.280^H^			6.184 ± 0.116^**ghi**^		

∗ Means ± Standard Error of means those do not share a letter are significantly different (P < 0.05) according to Tukey Method. Uppercase letters are used for “Weight of Fresh Buds” group and lowercase letters are used for “Weight of Made Tea” group.

After preparation of black tea, the highest amount of tea was obtained at radiation dose of 12 kGy, with the concentration of 300 ppm as expected. An average of 8.626 g of dry leaves were obtained, which is about 39.49 % higher than the untreated control and showed a significant difference at 95% confidence level. For 150 ppm ISAS treatment, the highest result was also obtained at 12 kGy. At 500 ppm concentration, it gave the best result when irradiated at 20 kGy.

#### 3.2.3 Correlation between weight of fresh leaves and made tea

**Figure 3:**
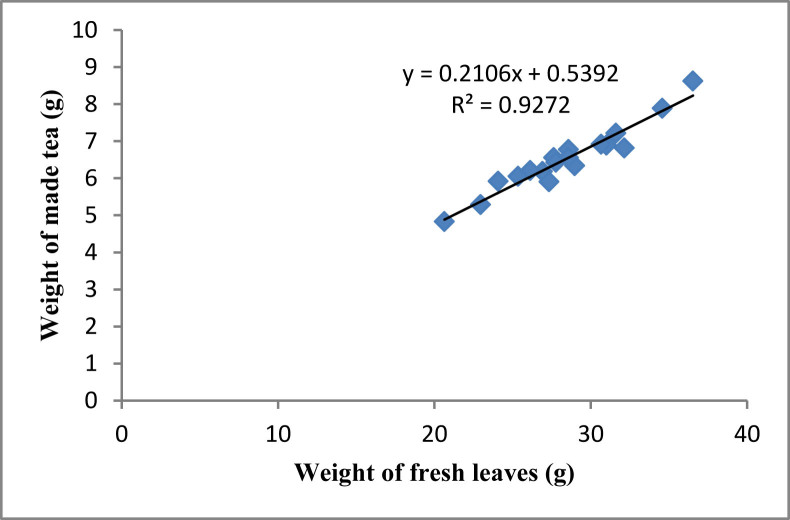
Correlation between weights of fresh leaves and made tea.

It is very important for efficient tea production that the made tea has the rational weight with fresh leaves. To rationalize the weight of made tea in terms of fresh weight of tea leaves, linear regression plot was generated and the correlation co-efficient were calculated (Figure 3). Overall, a striking correlation between fresh weight of tea leaves and weight of made tea was noted (r^2^ = 0.927). So, the weight of made tea was highly correlated with the weight of fresh leaves for all treatments.

#### 3.2.4 Average weight per bud

**Figure 4:**
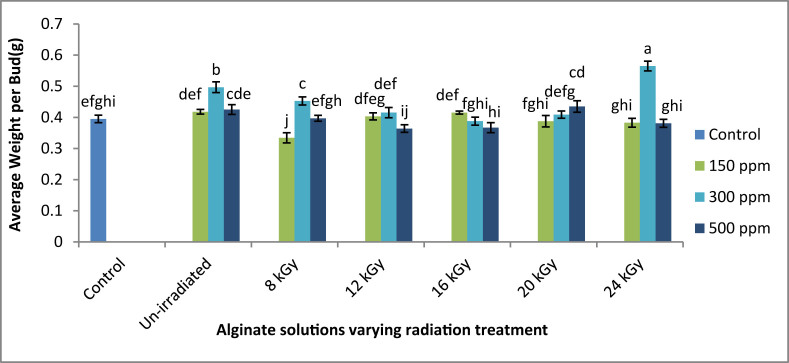
Effects of gamma radiation treated sodium alginate solutions on average weight per bud. **∗**Means ± Standard error of means those do not share a letter are significantly different (P < 0.05) according to Tukey Method.

The maximum weight of tea bud was obtained at 300ppm ISAS treatment when it was radiated at 24 kGy (Figure 4). It that case, about 37.16% increase in the average weight of bud was obtained in contrast with control plants. At 150 ppm concentration, maximum bud weight was also obtained when the solution was irradiated at 24 kGy. At 300ppm concentration, maximum average bud weight was obtained when the solution was irradiated at 20 kGy.

#### 3.2.5 Average leaf area

**Figure 5:**
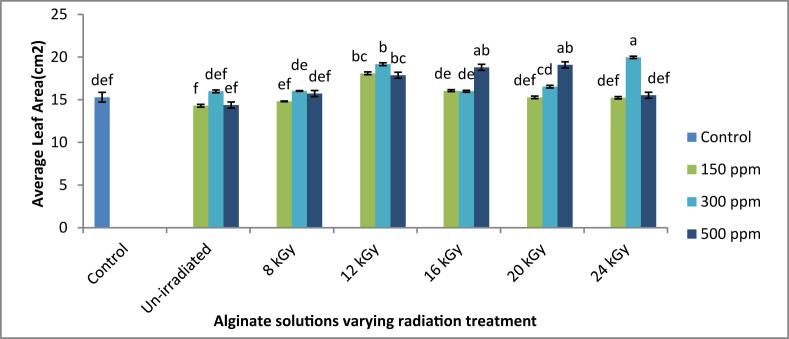
Effects of irradiated sodium alginate solutions on average leaf area. ∗Means ± Standard Deviation; those do not share a letter are significantly different (P < 0.05) according to Tukey Method.

Sodium alginate provided maximum leaf area at 24 kGy radiation doses, and at a concentration of 300 ppm as it was found for weight per bud (Figure 5). At this treatment, it provided average leaf area of 19.960 cm^2^, which was 30.52 % larger than the control (15.292 cm^2^). However, the bud number decreased at this treatment. For 150 ppm concentrated solution, highest result was obtained from 12 kGy radiation doses. At 500 ppm concentrated alginate solution, it gave the best result at 20 kGy radiation doses. But these values were not significantly different from the value of control.

### 3.3 Anti-fungal activity

**Figure 6:**
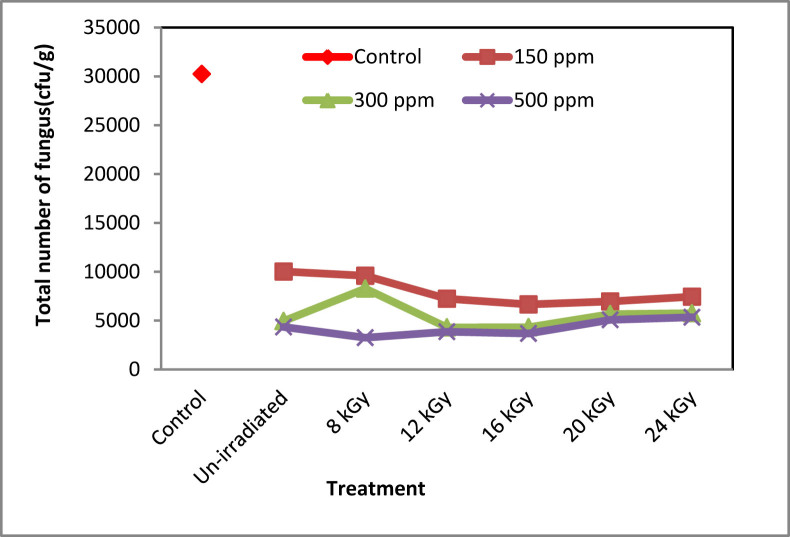
Anti-fungal activity of radiation processed sodium alginate solutions on tea plants.

As plants are very prominent habitat of fungus, so the total fungal count is one of the dominating factors for plants growth hence the tea productivity. It was found that, irradiated sodium alginate greatly affect the total fungal count of tea leaves (Figure 6). For 500 ppm concentrated sodium alginate irradiated at 12 kGy doses, the fungal count was reduced about 10 times than that of control plants. Colony forming units were found to be 30240 for controlled plants whereas the lowest number of fungal counts was 3250 (at 08 kGy, 500 ppm). The antifungal activity was found higher in more concentrated solutions.

## 4 Discussion

It has been demonstrated that several exogenous and endogenous factors regulate the growth, development and yield of a plant (Srivastava and Srivastava, 2007). Among exogenous factors, various plant growth promoters are known which have direct or indirect influence on growth of the plant. Hence, this study was aimed to demonstrate a comparative study between irradiated and non-irradiated sodium alginate as tea plant growth promoter. The very first noticeable observation was the drastic reduction in viscosity of the sodium alginate polymer upon Co-60 gamma ray treatment. As the molecular weights of polysaccharides are directly related to its viscosity (Huei and Hwa, 1996), the reduced viscosity suggested direct impact on the molecular weight of sodium alginate polymer. Similar observation on sodium alginate was also reported by Naeem et al. (2015). It was also reported that polysaccharides such as alginate, carrageenan and chitosan had novel properties like promotion of germination and shoot elongation in their depolymerized form (Taha and Sorour, 2019; Mirajkar et al., 2019; Idrees et al., 2011).

From this experiment, it was found that 12 kGy gamma irradiated 300 ppm sodium alginate solution had the best activity with respect to average buds' number, total bud weight and overall tea production. However, maximum average bud weight as well as maximum leaf area were found for the 300 ppm, 24 kGy ISAS treatment. On the other hand, un-irradiated alginate treatment showed moderate growth enhancement compared to untreated control plants. These results suggested that smaller alginate molecules had higher stomatal uptake and which stimulated the photosynthesis and thus showed enhanced leaf size. In addition to reducing the molecular weight of the sodium alginate, ionizing γ-irradiation also brings about certain structural reforms such as the introduction of double bonds, the development of increased polar end groups, etc., which cause sodium alginate to bind several receptor proteins leading to various signaling pathways, causing plant growth and yield. Sadiq et al. (2017) reported that the use of irradiated sodium alginate might have enhanced photosynthate's proficient assimilation and effective translocation, which could have contributed to improved leaf growth of the spearmint plant. Besides, Hien et al. (2000) disclosed the important role of degraded alginate in the induction of cell signaling in different plants, leading to the stimulation of different physiological processes and increased plants growth. According to El-Rehim et al. (2009), the absorption of sodium alginates served as a growth booster, and resulted in plant root and shoot elongation, thereby increasing plant production and enhancing physiological parameters in contrast with untreated plants. Taha and Sorour (2019) interpreted that irradiated sodium alginate caused an increase in the leaf area, which ascribed to use of additional CO_2_ by the leaves that improves photosynthesis and deposition of more dry matter while receiving more sunlight. Similar growth enhancements were also found for the ISAS treatment on various crops irrespective to plant categories e.g. fruit, vegetables, oil or legume producing plants (Uddin et at., 2020; Aftab et el., 2016; Naeem et el., 2015; Kume et al., 2002; Chmielewskia et al., 2007; and Albayrak, 2005).

In contrast, 12 kGy ISAS treatment showed slightly smaller leaf size compared to 24 kGy ISAS treatment but produced better leaves compared to all other treatments. These suggested that 12 kGy radiation treated sodium alginate might have the optimal molecular size and structural orientation for physiological processing, transport and bioactivity in tea plant. These results support the “specific structural and size requirement” hypothesis of Darvill et al. (1992) for inducing the physiological processes. Furthermore, antifungal studies suggested that although 12 kGy ISAS treatment didn't showed the best antifungal activity like 500 ppm, 8 kGy ISAS treatment (~10 fold less fungal count compared to untreated control), 300 ppm, 12 kGy ISAS treatment was also found very effective to reduce fungal load on tea plants (~8 fold less fungal count compared to untreated control). So, based on the experimental results, 300 ppm, 12 kGy ISAS treatment can be designated as the most effective treatment for boosting tea production. In case of 500 ppm treatment, the concentration alginate oligomers might be overdosed which downregulated the growth enhancement.

Overall, the absorption of alginate oligomers acted as a growth promoter, which resulted in plant root and shoot elongation and thereby led to increase in tea productivity. Furthermore, irradiated sodium alginate played a role of cell signaling in tea plant for the induction of phytoalexins which also played an important role for increased productivity (Sadiq et al., 2017). Dey et al. (2019) hypothesized that sodium alginate pretreatment aid in conversing disease resistance to tomato plants against *A*. *solani* by decreasing enzymatic activity of catalase and accumulating higher level of H_2_O_2_ at the site of infection.

## 5 Conclusion

The study had an opportunity to look into potential uses of sodium alginate as they are cheap, renewable, eco-friendly, non-harmful, and available in nature. Based on the present study, it can be concluded that radiation processed sodium alginate has progressive impact on tea plants in terms of productivity and fungus resistance. Based on the field trial, about 35.71% of increased tea productivity was found for the 300 ppm, 12 kGy irradiated alginate solution treatment. It was also found that un-irradiated as well as other formulations of alginate treatment had positive impact on the tea productivity. However, 300 ppm, 12 kGy treatment was found to be the best based on the overall effects. Here, it should be mentioned that although the experiment showed progressive effect on tea plants, the results were not in harmony for all treatments, which might arise due to seasonal or environmental factors.

## Declarations

### Author contribution statement

Mohammad Afzal Hossain: Performed the experiments; Wrote the paper.

Jahid M M Islam: Conceived and designed the experiments; Analyzed and interpreted the data; Wrote the paper.

Md. Mozammel Hoque: Analyzed and interpreted the data.

Shamsun Nahar: Performed the experiments.

Mubarak A Khan: Conceived and designed the experiments; Contributed reagents, materials, analysis tools or data.

### Funding statement

This research did not receive any specific grant from funding agencies in the public, commercial, or not-for-profit sectors.

### Data availability statement

Data included in article/supplementary material/referenced in article.

### Declaration of interests statement

The authors declare no conflict of interest.

### Additional information

No additional information is available for this paper.
